# SIPA1L3 via its PDZ domain inhibits the tight junction-associated AMOT-Patj to promote a malignant phenotype in NSCLC

**DOI:** 10.1097/MD.0000000000045095

**Published:** 2025-10-10

**Authors:** Lin Wang, Bin-Xue Wang, Rui Zhang, Si-Han Xian, Si Wang

**Affiliations:** a Blood Transfusion Department, National Cancer Center, National Clinical Research Center for Cancer, Cancer Hospital and Shenzhen Hospital, Chinese Academy of Medical Sciences and Peking Union Medical College, Shenzhen, Guangdong Province, PR China; b Department of Medical Microbiology and Human Parasitology, College of Basic Medical Sciences, China Medical University, Shenyang, PR China.

**Keywords:** AMOT, Hippo pathway, NSCLC, Patj, SIPA1L3

## Abstract

**Background:**

Signal-induced proliferation-associated 1 like 3 (SIPA1L3) is a member of the protein family. Very limited data are currently available regarding the role of SIPA1L3 in human carcinoma. Therefore, in this study, we investigated the expression pattern and function of SIPA1L3 in non-small cell lung cancer (NSCLC).

**Methods:**

We analyzed the distribution of SIPA1L3 in NSCLC specimens by immunohistochemistry, the relationship between SIPA1L3 expression and patient clinicopathological features, and investigated the effect of SIPA1L3 on cell growth and invasion in vivo and in vitro using small interfering RNA. Western blotting and immunoprecipitation were performed to demonstrate the interaction between SIPA1L3 and tight junction-associated angiomotin (AMOT) and Pals1-associtated tight junction protein.

**Results:**

We found that SIPA1L3 was overexpressed in NSCLC clinical tissue samples and was associated with several clinicopathological factors. SIPA1L3 affects the proliferation and invasion of cancer cells both in vivo and in vitro. Using a SIPA1L3 mutant, we found that SIPA1L3 interacts with AMOT through its PDZ domain, which inhibits the binding of AMOT to Pals1-associtated tight junction protein and further decreases AMOT anchoring to tight junctions.

**Conclusion:**

Our findings suggested that SIPA1L3 promotes tumorigenesis in lung cancer cells through its PDZ domain-mediated interaction with AMOT, suggesting that SIPA1L3 is a novel candidate gene that contributes to the malignant phenotype of lung cancer.

## 1. Introduction

Cancer recurrence and metastasis are the main causes of death in lung cancer patients. Although the risk factors for recurrence and metastasis have been extensively studied, the underlying genomic mechanisms remain largely unknown.^[1–3]^ Signal-induced proliferation-associated 1 like 3 (SIPA1L3) is a member of the signal-induced proliferation-associated (SIPA) protein family, which comprises 3 other members: SIPA1, SIPA1L1, and SIPA1L2.^[4–6]^ All SIPA proteins are expressed in the central nervous system, and most studies have focused on their functions within the brain. In addition, SIPA1L3 expression has been reported in the embryonic lens; consequently, mutations in the human SIPA1L3 gene result in congenital cataracts.^[7–9]^ Although studies have reported that SIPA1L3 plays an important role in epithelial cell morphogenesis, the establishment or maintenance of cell polarity, cell adhesion, and cytoskeletal organization, the functional role of SIPA1L3 in human carcinomas, including non-small cell lung cancer (NSCLC), remains unclear. Therefore, in the present study, we investigated whether SIPA1L3 was abnormally expressed in NSCLC and its role in tumorigenesis.

The full-length human SIPA1L3 protein consists of 1781 amino acid residues, with a predicted Rap GTPase-activating protein domain (residues 611–828), a PDZ domain (residues 966–1042), and a C-terminal coiled–coiled domain (residues 1720–1774). In the present study, we explored the possible regulatory role of the PDZ domain.

Tight junctions (TJ) are the most apical components of the junctional complex and provide a form of cell–cell adhesion in epithelial cells. The disruption of TJ function has been reported to be associated with tumorigenesis.^[10–12]^ Angiomotin (AMOT) is a scaffolding protein associated with adhesive contact between vertebrate epithelial cells. In epithelial cells, AMOT increases TJ integrity by recruiting and binding a variety of TJ proteins.^[13–15]^ AMOT, which has conserved C-terminal PDZ-binding motifs, has been reported to associate with TJ by binding to Pals1-associtated tight junction protein (Patj) and Pals1.^[16,17]^ Given that SIPA1L3 contains a PDZ domain that can bind to the PDZ binding motif, we wanted to explore whether SIPA1L3 interacts with AMOT and plays a functional role in TJ formation during lung cancer development.

This study aimed to explore the expression pattern of SIPA1L3 in NSCLC and its possible regulatory mechanisms, thereby providing novel insights into the molecular mechanisms of lung cancer recurrence and metastasis, and prevention and treatment of the same.

## 2. Materials and methods

### 2.1. Specimen collection

This study was approved by the Institutional Review Board of China Medical University (approval no. 2017025) and was conducted in accordance with the Declaration of Helsinki. Informed consent was obtained from all the patients for specimen analysis.

A total of 217 specimens, 114 and 103 from male and female patients with NSCLC, respectively, were obtained either by biopsy or complete resection at the First Affiliated Hospital of China Medical University between 2000 and 2012. According to the 2015 World Health Organization lung cancer histopathological diagnostic criteria, 134 cases were categorized as adenocarcinoma and 83 as squamous cell carcinoma, which included 117 well-differentiated, 72 moderately differentiated, and 28 poorly differentiated cases. The mean patient age was 57 years (range: 33–72 years). Lymph node metastases were identified in 92 of the 217 patients. According to the 80th edition of the American Joint Committee on Cancer Tumor Node Metastasis staging criteria published by the International Association for the Study of Lung Cancer in 2017, NSCLC specimens were classified as stage I (n = 123), II (n = 18), and III (n = 76). Follow-up information was obtained by reviewing patients’ medical records. None of the patients was treated with radiotherapy, chemotherapy, immunotherapy, or tyrosine kinase inhibitors before definitive pathological diagnoses were determined. Of the 217 samples, 40 fresh specimens, including both tumor and corresponding normal tissue samples, were stored at −70°C immediately after resection for protein extraction.

### 2.2. Immunohistochemistry (IHC) staining

Briefly, tissue sections were incubated with normal goat serum to block nonspecific antibody binding, followed by incubation with a rabbit polyclonal anti-SIPA1L3 antibody (ab113657, 1:300; Abcam, Cambridge, UK) and a secondary antibody (ab205718, 1:1000; Abcam). Positive SIPA1L3 expression was determined based on the intensity of cytoplasmic staining of tumor cells and the proportion of SIPA1L3-positive cells in the tissue sections. Two independent pathologists evaluated the staining in a blinded manner by randomly selecting 5 fields of view per slide at 400× magnification, with each field of view containing at least 100 cells. The following immunostaining assessment scale was used: negative, no staining; weak, light-yellow staining, no clear granular or yellow staining, and clear granular staining area <10%; moderate, yellow staining and clear granular staining area ≥10% or brown staining and clear granular staining area <10%; and strong, brown staining and clear granular staining area ≥10%. Negative to weak SIPA1L3 expression was considered low SIPA1L3 expression, whereas moderate to strong SIPA1L3 expression was considered SIPA1L3 overexpression.

### 2.3. Western blot analysis

The Protein concentration was determined using a bicinchoninic acid kit (Pierce, Rockford). Equal amounts of lysate were resolved using sodium dodecyl sulfate-polyacrylamide gel electrophoresis. The following primary antibodies were used: AMOT (B-4; sc-166924, 1:300), cyclin D (sc-8396, 1:200), cyclin E (sc-377100, 1:200), cyclin-dependent kinase 4 (sc-23896, 1:300), CDK6 (sc-7961, 1:300), matrix metalloproteinase 2 (MMP2; sc-13595, 1:300), and MMP9 (sc-393859, 1:300) from Santa Cruz Biotechnology (Santa Cruz); SIPA1L3 (1:500) from Abcam; Myc Tag mouse monoclonal antibody (AF0033, 1:500) and green fluorescent protein Tag mouse monoclonal antibody (AF2882, 1:500) from Beyotime Biotechnology (Shanghai, China); glyceraldehyde 3-phosphate dehydrogenase (#5174, 1:10,000) from Cell Signaling Technology (cell signaling technology, Danvers); and Patj polyclonal antibody (PA5-85444, 1:1000) from Invitrogen (Waltham). Secondary antibodies were purchased from cell signaling technology (7074S, 1:1000). Protein expression was quantified using densitometry and ImageJ software, and protein bands were visualized using ECL western blotting substrate (Pierce) and a BioImaging System (UVP Inc., Upland). GAPDH was used as an internal control for normalization.

### 2.4. Cell lines and transfection

Human bronchial epithelial cells, human adenocarcinoma cell lines (A549, LTEP-a-2, H1299), and squamous cell carcinoma cell lines (LK-2 and SK-MES-1) were purchased from the American Type Culture Collection (Manassas; The cell lines were obtained in February 2017 and tested by STR, basing on plate gel and capillary electrophoresis analysis. The last test date was July 10, 2022). SIPA1L3-small interfering RNA (siRNA), SIPA1L3-cDNA (with Myc tag), SIPA1L3-ΔPDZ (position 966–1042)-cDNA (with Myc tag), Patj-cDNA (with GFP tag), and negative controls were purchased from GeneChem Biological Technology Co., Ltd. (Shanghai, China). Cells were transfected with plasmids using Attractene Transfection Reagent (Qiagen, Hilden, Germany) according to the manufacturer’s instructions.

### 2.5. Immunofluorescence staining

Cells were fixed, permeabilized, and incubated with primary antibodies against SIPA1L3 and AMOT and subsequently with fluorescein isothiocyanate-conjugated or tetramethylrhodamine isothiocyanate-conjugated secondary antibodies (A0423, A0453, Beyotime Biotechnology). Nuclei were stained with 4′,6-diamidino-2-phenylindole, and cells were observed using a confocal microscope (Carl Zeiss, Olympus, Tokyo, Japan).

### 2.6. CCK-8 assay

A CCK-8 kit (GlpBio Technology, Montclair) was used to measure cell proliferation, according to the manufacturer’s instructions. A total of 1000 cells per 100 μL were cultured per well in a 96-well plate. Then, 10 μL of the CCK-8 reagent was added to each well. The assay was performed at 0, 24, 48, 72, 96, and 120 hours. At each time point, the OD450 was determined using a microplate reader (Bio-Rad Laboratories, Hercules).

### 2.7. Matrigel invasion assay

The cell invasion assay was performed in a 24-well transwell chamber with an 8-μm pore size (Costar, Cambridge). The transwell inserts were coated with 20 μL Matrigel (1:3 dilution; BD Biosciences, Franklin Lakes). Forty-eight hours after transfection, 3 × 10^5^ cells in 100 μL of serum-free medium were seeded in the upper chamber of the Matrigel-coated Transwell insert and incubated for 16 hours, and 600 μL of culture medium supplemented with 10% fetal bovine serum was added to the lower chamber. After 16 hours of incubation, the non-invading cells were removed from the upper membrane using a cotton swab, and the cells that passed through the filter were fixed with 4% paraformaldehyde and stained with hematoxylin. The number of invading cells was counted in 10 randomly selected high-power fields under a microscope. This experiment was performed in triplicates.

### 2.8. Colony formation assay

Cells were plated into 6-cm cell culture dishes at a density of 1000 cells per dish and incubated for 14 days. The plates were washed with phosphate buffered saline (PBS), fixed with 4% paraformaldehyde, and stained with Giemsa stain. Colonies containing >50 cells were counted under a microscope. Images were obtained and the number of colonies was counted manually.

### 2.9. Xenograft tumorigenicity assay

The mice were treated according to the experimental animal ethics rules of China Medical University. This study was approved by the Laboratory Animal Welfare and Ethical Review Committee of the China Medical University (approval no. CMU2021099). Four-week-old (16–20 g) male BALB/c nude mice (specific pathogen-free standard) were purchased from the Beijing Charles River Company (Beijing, China) and housed in a specific pathogen-free environment at a temperature of 20 to 25°C and relative humidity of 40% to 70%. Food and drinking water were sterilized using a semi-barrier system at a constant temperature and humidity. Each nude mouse was randomly assigned to the control siRNA and SIPA1L3 siRNA groups (n = 7/group). The investigators were blinded to the allocation.

The cell concentration for each group was adjusted to 5 × 10^6^ cells/mL in PBS, and 200 μL of the cell suspension was injected subcutaneously into the backs of the nude mice. After 4 weeks, the mice were sacrificed and the weights and volumes of the subcutaneous tumors were recorded. Tumor samples were analyzed using western blotting and IHC.

### 2.10. Co-immunoprecipitation (Co-IP)

Cell lysates were centrifuged at 14,000×g and the protein concentration was determined using a BCA kit (Pierce). Equal amounts of cell lysate (400 μg) were divided into input, IgG, and IP groups and then incubated with protein A+ G agarose (20 μL; Beyotime Biotechnology) and IgG (1 μg, Beyotime Biotechnology) for 2 hours at 4°C to block nonspecific binding. After centrifugation at 1000×g for 120 hours, the supernatant was collected and incubated overnight at 4°C with 2 μg of rabbit AMOT (Santa Cruz Biotechnology) or control rabbit IgG (Beyotime Biotechnology). Subsequently, protein A + G agarose (20 μL) was added to the supernatants, which were further incubated for 3 hours at 4°C. After centrifugation at 1000×g, the beads were collected and washed thrice with cold PBS. The samples were subjected to western blotting using the anti-Myc, GFP, AMOT, SIPA1L3, and Patj primary antibodies. The experiment was repeated twice and the input group was used as an internal control for normalization.

### 2.11. Statistical analysis

SPSS (version 24.0; IBM Corp., Armonk) was used for all statistical analyses. Correlations between SIPA1L3 expression and clinicopathological parameters of the patients were assessed using the chi-square test. Survival curves were generated using the Kaplan–Meier method. Each experiment involving the cell lines was performed at least thrice. Western blot gray values were detected using Image LabTM (Bio-Rad Laboratories) and compared using the Student *t* test. Statistical significance was set than .05 (2-sided) indicated significance. Columns represent mean values and bars represent SD.

## 3. Results

### 3.1. Overexpression of SIPA1L3 in patients with NSCLC is correlated with a malignant phenotype

**Figure 1.**
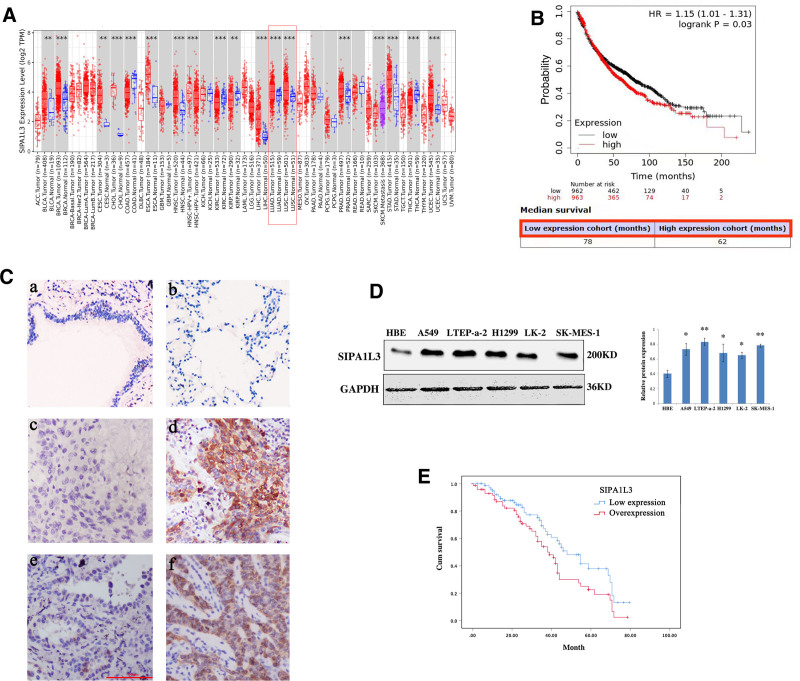
Signal-induced proliferation-associated 1-like protein 3 (SIPA1L3) showed overexpression in non-small cell lung cancer tissues. We used the cancer genome atlas (TCGA) database (https://genome-cancer.ucsc.edu/) to predict SIPA1L3 mRNA expression (A) and the association between its expression and the overall survival of patients with lung carcinomas. (B) n = 2437; the cutoff value used in the analysis was 353, and the expression range of the probe was 16–2976. (C) Immunohistochemistry analysis revealed negative SIPA1L3 staining in the normal bronchial epithelium (a), alveolar epithelium (b), and well-differentiated lung squamous cell carcinoma (c). Cytoplasmic accumulation of SIPA1L3 is observed in moderately and poorly differentiated adenocarcinoma (d). (e) Well-differentiated adenocarcinoma showed lower expression of SIPA1L3. Cytoplasmic accumulation of SIPA1L3 was observed in moderately and poorly differentiated adenocarcinomas (f). Bar, 100 μm. (D) Increased SIPA1L3 expression was detected in lung cancer cells compared to immortalized bronchial epithelial HBE cells (n ≥ 3; A549, *P* = .029; LTEP-a-2, *P* = .011; H1299, *P* = .032; LK-2, *P* = .035; and SK-MES-1, *P* = .014). (E) Kaplan–Meier curves for survival analysis of 155 patients in the present study for whom follow-up information was available. Patients with SIPA1L3 overexpression (73 cases) had poorer OS than those with low SIPA1L3 expression (82 cases; *P* = .026). Data are presented as mean ± SD. *P < .05, **P < .01, ***P < .001, by Student *t* test. SIPA1L3 = signal-induced proliferation-associated 1-like protein 3, TCGA = the cancer genome atlas.

The Cancer Genome Atlas database was used to analyze the SIPA1L3 mRNA expression patterns in lung cancer tissues. SIPA1L3 mRNA expression was dramatically higher in lung cancer tissues than in normal tissues (Fig. 1A). Additionally, patients with lung cancer and high SIPA1L3 levels showed poorer overall survival (Fig. 1B). Next, we performed IHC analysis on 217 NSCLC tissues. SIPA1L3 was undetectable in normal bronchial epithelia or pneumocytes; however, 147 out of 217 (67.74%) NSCLC specimens showed increased cytoplasmic expression of SIPA1L3 (Fig. 1C). Western blot analysis (Fig. 1D) was performed on lung cancer cell lines, and the results were consistent with those of the IHC analysis. To evaluate the clinical significance of SIPA1L3 in NSCLC tissues, we analyzed the correlation between SIPA1L3 overexpression and clinicopathological parameters. As summarized in Table 1, tumors with increased SIPA1L3 expression tended to display more malignant phenotypes such as poor differentiation (*P* = .016), higher TNM stage (*P* = .01), and positive lymphatic metastasis (*P* = .004). In terms of survival, complete follow-up information was available for 155 of 217 patients. Patients with SIPA1L3 overexpression had poorer overall survival than those with low SIPA1L3 expression (*P* = .026; Fig. 1E). These results suggest that SIPA1L3 acts as a tumor promoter in NSCLC.

**Table 1:** Overexpression of SIPA1L3 correlated with malignant phenotype in NSCLC patients.

Clinicopathological feature	Total N (217)	Overexpression N (147)	*ϰ* ^2^	*P*-value
Age (yr)				
<57	95	62 (65.26%)	0.475	.491
≥57	122	85 (69.67%)		
Gender				
Male	114	79 (69.30%)	0.266	.606
Female	103	68 (66.02%)		
Histological type				
Squamous cell carcinoma	83	53 (63.86%)	0.929	.335
Adenocarcinoma	134	94 (70.15%)		
Differentiation				
Well	117	71 (60.68%)	5.788	.016
Moderate and poor	100	76 (76.00%)		
TNM classification				
I + II	141	87 (61.70%)	6.721	.010
III	76	60 (78.95%)		
Lymph node metastasis				
Positive	92	72 (78.26%)	8.087	.004
Negative	125	75 (60.00%)		

NSCLC = non-small cell lung cancer, SIPA1L3 = signal-induced proliferation-associated 1-like protein 3, TNM = tumor node metastasis.

### 3.2. Downregulation of SIPA1L3 by siRNA inhibits lung cancer cell proliferation and invasion in vitro and in vivo

**Figure 2.**
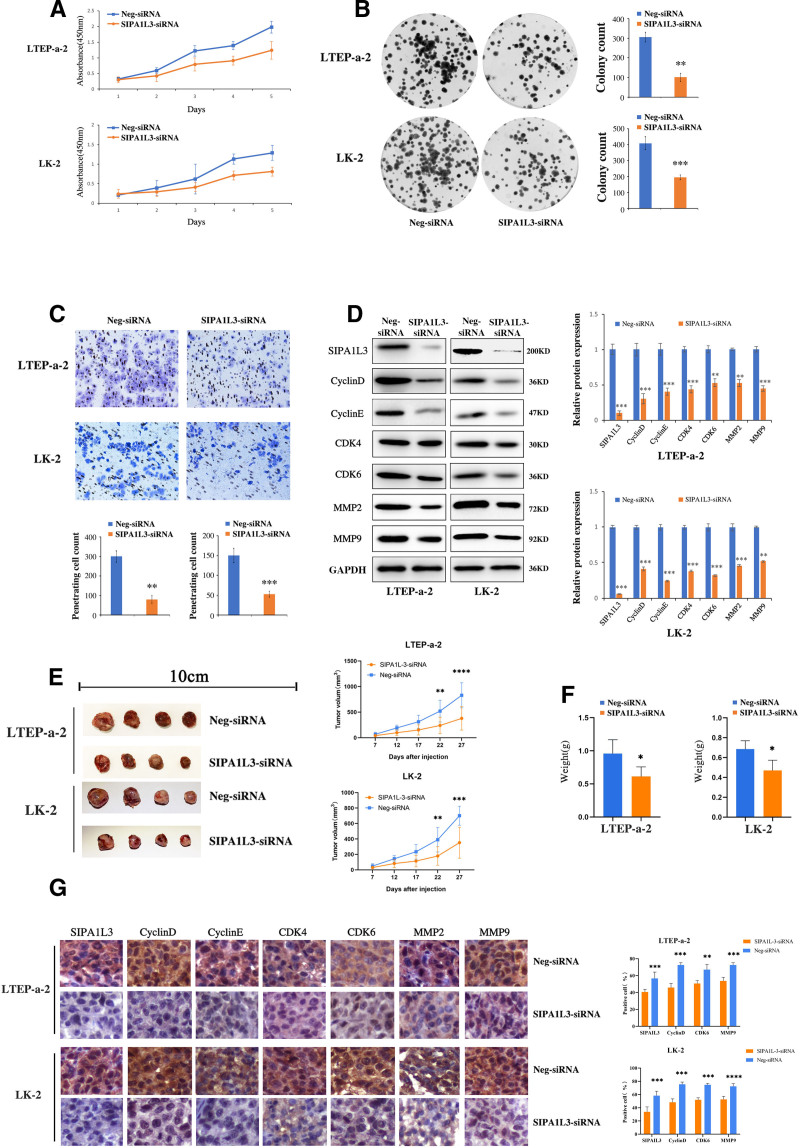
Downregulation of SIPA1L3 inhibited malignant phenotype of lung cancer cells in vivo and in vitro. (A) MTT assay was used to assess cell proliferation following transfection of LTEP-a-2 and LK-2 cells with the SIPA1L3-siRNA plasmid. SIPA1L3-siRNA transfected-LTEP-a-2 and LK-2 cells showed a time-dependent decrease in cell proliferation compared to the control cells. (B) A significant decrease in colony numbers was observed in SIPA1L3-siRNA transfected-LTEP-a-2 and LK-2 cells compared with the control cells (n ≥ 3). (C) The Transwell assay showed that invasion capacity was significantly inhibited after SIPA1L3-siRNA transfection (n ≥ 3). (D) The expression of cell proliferation-associated proteins including cyclin D, cyclin E, cyclin dependent kinase 4 (CDK4), and CDK6 and invasion-associated proteins including Rac1, Cdc42, matrix metalloproteinase 2 (MMP2), and MMP9 in lung cancer cell lines was analyzed by western blotting. SIPA1L3-siRNA transfection resulted in significant downregulation of the expression of cell proliferation- and invasion-associated proteins (n ≥ 3). The volumes (E) and weights (F) of subcutaneous transplanted tumors in the SIPA1L3-siRNA group were significantly reduced compared to that of the control group (n = 4). (G) The subcutaneous transplanted tumors in the SIPA1L3-siRNA group showed significant decrease in the expression of tumorigenesis-associated proteins (n ≥ 3). Data are presented as mean ± SD. *P < .05, **P < .01, ***P < .001, by Student *t* test. CDK4 = cyclin dependent kinase 4, MMP2 = matrix metalloproteinase 2, SIPA1L3 = signal-induced proliferation-associated 1-like protein 3, siRNA = small interfering RNA.

Transfection of LTEP-a-2 and LK-2 cells with SIPA1L3-siRNA decreased SIPA1L3 expression, which in turn inhibited cell growth (Fig. 2A). As shown in Figure 2B, SIPA1L3 knockdown considerably reduced the number and size of the lung cancer cell colonies. In addition, SIPA1L3 knockdown considerably reduced cell invasion compared to that in the control cells (Fig. 2C). Therefore, the expression of cell proliferation- and invasion-associated proteins was analyzed using western blotting. As shown in Figure 2D, the expression of cyclin D, cyclin E, cyclin-dependent kinase 4, and CDK6 was significantly downregulated in the SIPA1L3-siRNA-transfected LTEP-a-2 and LK-2 cells. Additionally, the expression of invasion-associated proteins MMP2 and MMP9 was considerably downregulated.

To further verify the effect of SIPA1L3 on cell proliferation in vivo, we performed a subcutaneous tumor transplantation in nude mice. Compared with the control group, the volumes (Fig. 2E) and weights (Fig. 2F) of subcutaneous tumors in the SIPA1L3-siRNA group were significantly decreased compared to that in the control group. Additionally, the expression of cell proliferation- and invasion-associated proteins was lower in subcutaneous tumors from the SIPA1L3-siRNA group than in tumors from the control group (Fig. 2G). These findings are consistent with the results of in vitro experiments. Collectively, these results confirmed that reduced SIPA1L3 expression inhibited the malignant phenotype of lung cancer cells.

### 3.3. The PDZ domain of SIPA1L3 is responsible for SIPA1L3-mediated regulation of the expression of proliferation- and invasion-associated proteins

**Figure 3.**
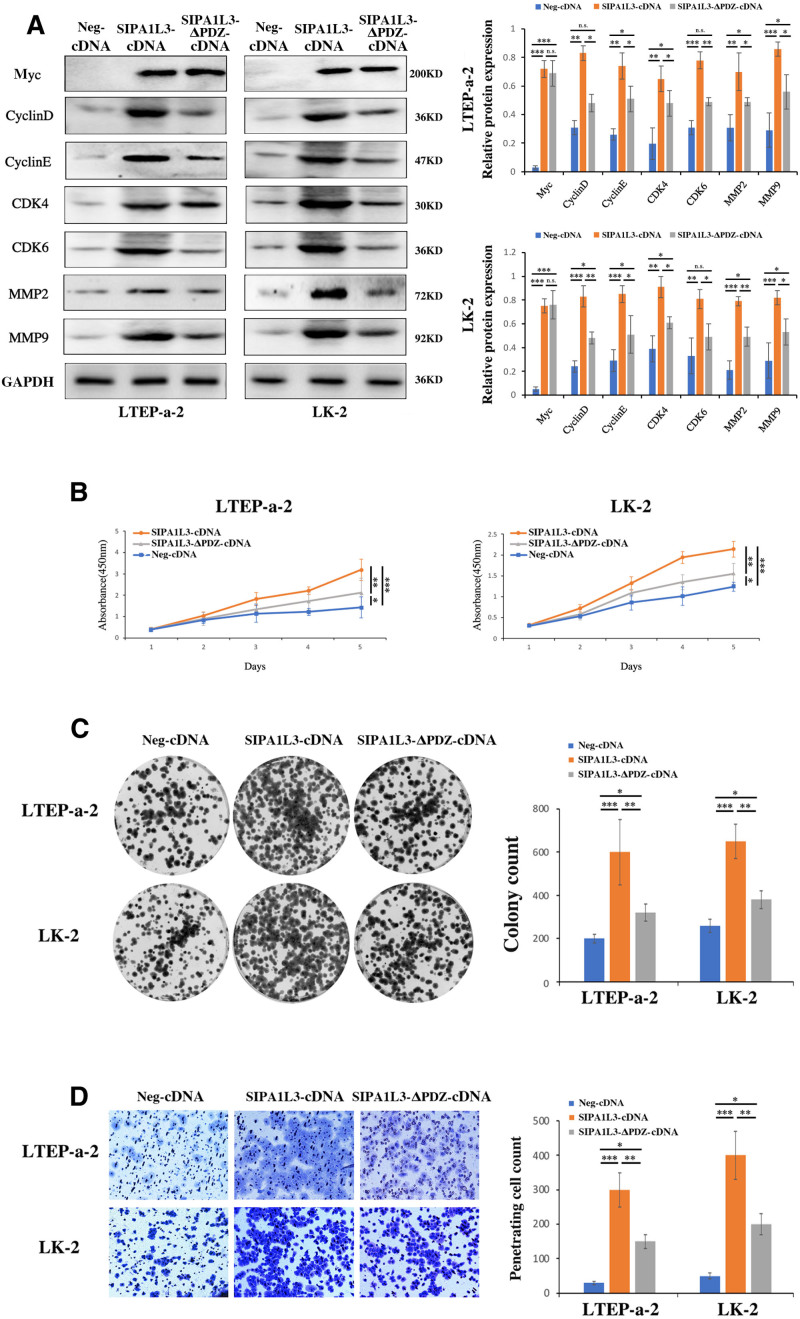
PDZ domain of SIPA1L3 regulates proliferation and invasion in lung cancer cells. (A) Western blot showed that SIPA1L3 overexpression significantly increased the expression of cyclin D, cyclin E, CDK4, CDK6, Rac1, Cdc42, MMP2, and MMP9. However, transfection of the SIPA1L3 mutant downregulated the expression of proliferation-associated and invasion-associated proteins (n ≥ 3). MTT (B), colony formation assay (C), and transwell assay (D) showed that overexpression of SIPA1L3 significantly increased proliferation and invading ability, whereas transfection of SIPA1L3 mutant decreased cell proliferation and invading ability (n ≥ 3). Data are presented as mean ± SD. *P < .05, **P < .01, ***P < .001, by Student *t* test. CDK4 = cyclin dependent kinase 4, MMP2 = matrix metalloproteinase 2, SIPA1L3 = signal-induced proliferation-associated 1-like protein 3.

To explore the possible role of the PDZ domain of SIPA1L3 in regulating tumorigenesis in lung cancer, the expression of important proliferation- and invasion-associated proteins was analyzed in cells overexpressing SIPA1L3 and in cells expressing SIPA1L3 mutants without the PDZ domain. As shown in Figure 3A, the expression of proliferation- and invasion-associated proteins was considerably higher in cells overexpressing SIPA1L3 than in the control cells. In contrast, cells with the SIPA1L3 mutant did not exhibit significantly higher expression of invasion-associated proteins. Overexpression of SIPA1L3 considerably promoted cell growth and invasion, whereas the SIPA1L3 mutant promoted limited cell growth and invasion (Fig. 3B–D). Taken together, these results illustrate that SIPA1L3 promotes the malignant phenotype of lung cancer cells via its PDZ domain.

### 3.4. SIPA1L3 interacts with AMOT and inhibits the binding of AMOT with Patj

**Figure 4.**
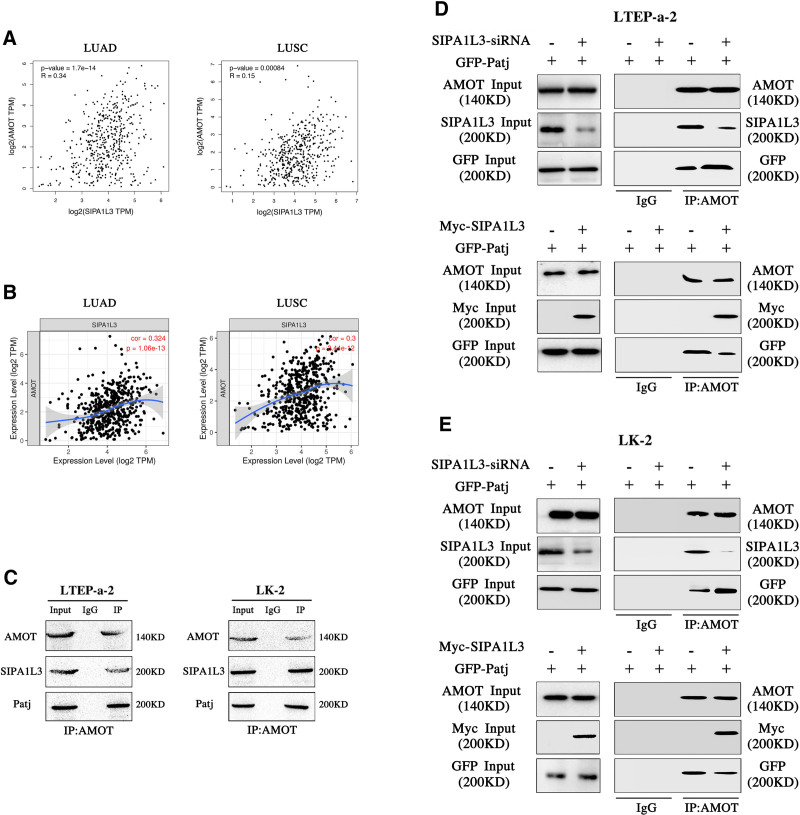
SIPA1L3 interacts with AMOT and inhibits the interaction of AMOT with Patj. Gene expression profiling interactive analysis (A) and tumor immune estimation resource (B) databases showed a positive correlation between SIPA1L3 and AMOT expression. Endogenous AMOT interacts with SIPA1L3 and Patj (C). Co-immunoprecipitation showed that the knockdown of SIPA1L3 in LTEP-a-2 cells inhibited its binding to AMOT and enhanced the binding of AMOT and Patj. Conversely, SIPA1L3 overexpression increased its interaction with AMOT and decreased its binding to Patj (D). LK-2 cells showed similar results to those of LTEP-a-2 cells (E). AMOT = angiomotin, Patj = Pals1-associtated tight junction protein, SIPA1L3 = signal-induced proliferation-associated 1-like protein 3.

Using the Gene Expression Profiling Interactive Analysis (Fig. 4A) and Tumor Immune Estimation Resource (Fig. 4B) databases, we observed a positive correlation between SIPA1L3 and AMOT. Co-IP confirmed that endogenous SIPA1L3 and AMOT interacted. In addition, an interaction between endogenous AMOT and Patj was observed in the lung cancer cells (Fig. 4C). After increasing the expression of exogenous Patj, SIPA1L3 knockdown decreased the binding of AMOT to SIPA1L3, resulting in increased binding of AMOT to Patj. Conversely, SIPA1L3 overexpression increased its interaction with AMOT and decreased the binding between AMOT and Patj (Fig. 4D, E).

### 3.5. The PDZ domain of SIPA1L3 mediates its interaction with AMOT and regulates cellular localization of AMOT

**Figure 5.**
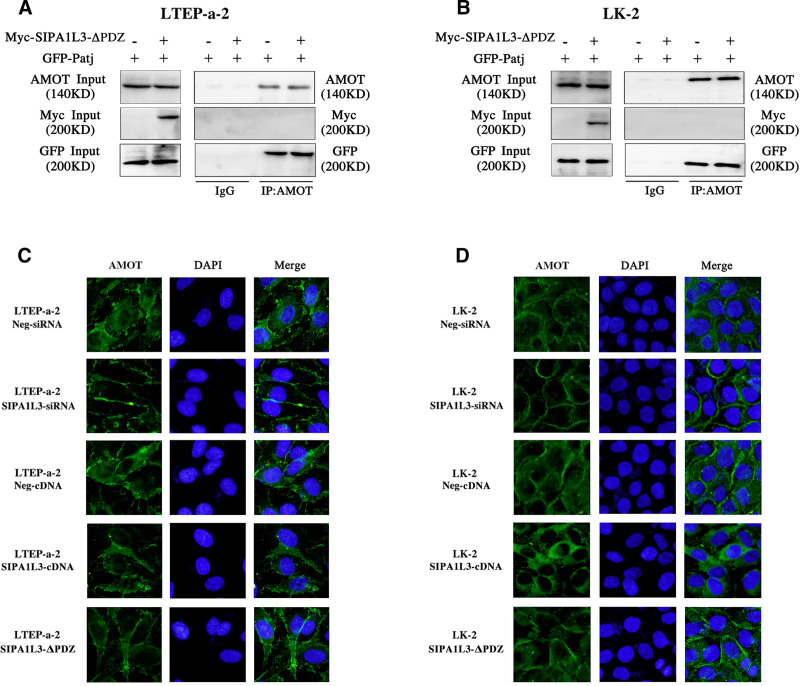
PDZ domain of SIPA1L3 mediates its binding to AMOT and regulations cellular localization of AMOT. (A, B) SIPA1L3 mutant does not interact with AMOT and does not affect the interaction between AMOT and Patj. (C, D) Downregulation of SIPA1L3 increased the membranous expression of AMOT, whereas overexpression of SIPA1L3 increased the cytoplasmic expression of AMOT. However, the SIPA1L3 mutant did not have an effect on AMOT localization. AMOT = angiomotin, Patj = Pals1-associtated tight junction protein, SIPA1L3 = signal-induced proliferation-associated 1-like protein 3.

We transfected lung cancer cell lines with an SIPA1L3 mutant plasmid lacking the PDZ domain. The SIPA1L3 mutant did not interact with AMOT; however, there was no change in the interaction between AMOT and Patj (Fig. 5A, B). Immunofluorescence results showed that knockdown of SIPA1L3 increased membranous expression of AMOT, whereas overexpression of SIPA1L3 increased cytoplasmic expression of AMOT. However, SIPA1L3 mutation had no effect on the cellular localization of AMOT (Fig. 5C, D). These findings suggested that the PDZ domain of SIPA1L3 mediates its binding to AMOT and regulates its translocation from the membrane to the cytoplasm in lung cancer cells.

## 4. Discussion

Previous reports on the expression of SIPA1L3 during embryogenesis and in some diseases provide a good rationale for further functional studies on SIPA1L3. Here, we analyzed the expression and significance of SIPA1L3 in lung cancer.

First, we examined the subcellular localization of endogenous SIPA1L3 in the lung tissues using IHC staining. SIPA1L3 was weakly detectable or undetectable in the normal bronchial epithelium, whereas 67.74% of the examined NSCLC tissues showed elevated SIPA1L3 expression, mainly in the cytoplasm. Clinically, a direct correlation was observed between upregulated cytoplasmic SIPA1L3 expression and advanced TNM stage, lymph node status, and poor differentiation. Following the evaluation of SIPA1L3 expression patterns in NSCLC, we explored the role of SIPA1L3 in regulating NSCLC cell proliferation and invasion in lung cancer cell lines. Knockdown of SIPA1L3 in LTEP-a-2 and LK-2 cells resulted in a significant decrease in cell proliferation and invasion capacity, as well as in the expression of proliferation- and invasion-associated proteins, both in vivo and in vitro. These results suggest a tumor-promoting function of SIPA1L3 in NSCLC.

We further investigated possible mechanisms underlying the role of SIPA1L3 in cell proliferation and invasion. Based on the predicted domain structure, SIPA1L3 possesses an N-terminal PDZ domain that is predicted to be required for protein–protein interactions. Hence, we explored whether the PDZ domain of SIPA1L3 has a regulatory effect on the malignant phenotype of cells by transfecting lung cancer cells with a SIPA1L3 mutant lacking the PDZ domain. We found that although the SIPA1L3 mutant can regulate cell proliferation and invasion, compared with full-length SIPA1L3, its regulatory role is limited. These findings suggest that SIPA1L3 promotes tumorigenesis in lung cancer cells via its PDZ domain.

TJ are cellular structures located in the apicobasal region of the epithelial cell membranes. TJ are the most apical components of the junctional complex and provide a form of cell–cell adhesion in epithelial cells.^[10,11]^ Disruption of TJ is well documented during cancer progression including in lung cancer.^[12,18,19]^ AMOT, a multifunctional scaffolding protein, has been shown to be localized at TJ, where it regulates cell polarity and adhesive contacts between vertebrate epithelial cells.^[13–15]^ AMOT exists as 2 isoforms, AMOT80 (~80 kDa) and AMOT130 (~130 kDa), which are generated by alternative splicing. AMOT has a coiled-coil domain and a conserved C-terminal PDZ-binding motif that allows it to interact with TJ-associated proteins. The isoform AMOT130 contains a unique N terminus extension.^[20,21]^

In mammalian epithelia, Patj and Pals1 are necessary for TJ integrity.^[22–25]^ AMOT has been reported to interact with Patj through its PDZ-binding motif, which targets AMOT to TJ, thereby recruiting it to the TJ complex. Given that SIPA1L3 possesses a PDZ domain, we explored the possible interaction between SIPA1L3 and AMOT130 in the present study. Bioinformatics analysis showed that SIPA1L3 correlated with AMOT in lung cancer. Using immunofluorescence and co-IP, we found that both SIPA1L3 and AMOT were mainly localized in the cytoplasm and interacted with each other. Additionally, we confirmed the interaction between AMOT and Patj through the PDZ-binding motif of AMOT. Therefore, we speculate that SIPA1L3 interacts with AMOT and competitively inhibits its binding to Patj.

We found that knockdown of SIPA1L3 inhibited its interaction with AMOT and enhanced the interaction of AMOT with Patj. In contrast, overexpression of SIPA1L3 enhanced its interaction with AMOT and consequently inhibited the binding of AMOT with Patj. To confirm that the PDZ domain of SIPA1L3 mediates the interaction between SIPA1L3 and AMOT, we transfected lung cancer cells with an SIPA1L3 mutant lacking the PDZ domain. Consistently, we observed that the SIPA1L3 mutant did not interact with AMOT or affect the interaction between AMOT and Patj, thereby confirming our hypothesis. Using immunofluorescence, we found that overexpression of SIPA1L3 prevented the membranous translocation of AMOT, whereas transfection of the SIPA1L3 mutant prompted AMOT localization to the membrane. These results showed that SIPA1L3, through its PDZ domain, binds to AMOT and competitively inhibits the binding of AMOT to the junctional protein Patj, thereby preventing the recruitment of AMOT to the cell membrane.

In epithelial cells, AMOT, via its coiled-coil domain or N-terminal extension, binds to a variety of different proteins, including enzymes, filamentous-actin, signaling effectors, and Rho guanosine triphosphatases and their regulators.^[16,26–30]^ Subsequently, AMOT recruits these proteins to TJ by binding to the PDZ-binding motif of Patj. For example, AMOT binds to Rich1 (a GTPase-activating protein for Cdc42) via a coiled-coil domain. The PDZ-domain binding motif of AMOT interacts with Patj and targets AMOT at the TJ containing Patj and Pals1. Therefore, Rich1, via its interaction with AMOT, is recruited to the TJ protein complex, where it plays a role in the maintenance of TJ integrity by the coordinated regulation of Cdc42 and links specific components of the TJ to intracellular protein trafficking.^[17]^

In addition, there are still areas in our research that require further improvement. Although we have demonstrated that AMOT can bind to SIPA1L3 through the PDZ domain of SIPA1L3, the coiled-coil domain in AMOT can also combine with the coiled-coil domain in SIPA1L3.^[31,32]^ We need to further prove that only when the PDZ domain in SIPA1L3 binds to the PB domain in AMOT can it inhibit the interaction between AMOT and Patj and recruit it to the TJ, while the combination of the coiled-coil domains in the 2 proteins cannot achieve this effect.

In summary, the analysis of clinical tumor samples in the present study strongly supported the notion that SIPA1L3 acts as a tumor oncogene in NSCLC. Our results demonstrate that SIPA1L3 increases cell proliferation and invasion through its PDZ domain by binding to AMOT, which inhibits the interaction of AMOT with Patj, further inhibiting the interaction of AMOT with its target proteins at TJ. The disruption of TJ function ultimately facilitates the malignant phenotype of lung cancer cells. Although there is limited understanding of the role of SIPA1L3 in cancer tumorigenesis, our findings suggest that overexpression of SIPA1L3 may be a marker for advanced NSCLC, providing new insights that can improve our understanding of the development of malignant lung cancer phenotypes.

## Acknowledgments

Special thanks go to our colleagues in the research group for their collaborative efforts and fruitful discussions. Their suggestions and contributions have enriched the content of this study.

## Author contributions

**Conceptualization:** Lin Wang, Si Wang.

**Data curation:** Lin Wang, Bin-Xue Wang, Rui Zhang, Si-Han Xian.

**Formal analysis:** Lin Wang, Bin-Xue Wang, Rui Zhang, Si-Han Xian.

**Funding acquisition:** Lin Wang, Si Wang.

**Investigation:** Bin-Xue Wang.

**Methodology:** Lin Wang, Rui Zhang.

**Project administration:** Si Wang.

**Resources:** Si Wang.

**Supervision:** Si Wang.

**Validation:** Rui Zhang, Si-Han Xian.

**Visualization:** Lin Wang, Bin-Xue Wang, Rui Zhang, Si-Han Xian.

**Writing** – **original draft:** Lin Wang.

**Writing** – **review & editing:** Si Wang.
